# Narrative Analyses: Cognitive Behavior Group Therapy for Women with Menopause and Bipolar or Major Depressive Disorders

**DOI:** 10.1089/whr.2021.0025

**Published:** 2021-09-22

**Authors:** Danette Conklin, Janet S. Carpenter, Meredith Sorenson Whitney, Sarah DeLozier, Daisy Okwa Ogede, Corinne Bazella, Molly McVoy, Martha Sajatovic

**Affiliations:** ^1^ Department of Psychiatry, University Hospitals Cleveland Medical Center, Case Western Reserve University, Cleveland, Ohio, USA.; ^2^ School of Nursing, Indiana University, Indianapolis, Indiana, USA.; ^3^ Department of Psychiatry, University Hospitals Cleveland Medical Center, Cleveland, Cleveland, Ohio, USA.; ^4^ Clinical Research Center, University Hospitals Cleveland Medical Center, Cleveland, Ohio, USA.; ^5^ Case Western Reserve University School of Medicine, Cleveland, Ohio, USA.; ^6^ Department of Obsterics and Gynecology, University Hospitals Cleveland Medical Center, Cleveland, Ohio, USA.; ^7^ Department of Psychiatry, University Hospitals Cleveland Medical Center, Case Western Reserve University School of Medicine, Cleveland, Ohio, USA.

**Keywords:** bipolar disorder, major depressive disorder, menopause, cognitive behavioral group therapy, content analysis

## Abstract

***Background:*** Bipolar and depressive disorders (bipolar disorder [BD], major depressive disorder [MDD]), as well as menopause affect millions of women. Although there are three known cognitive behavioral group treatment (CBGT) protocols to help women with problematic menopause symptoms, they do not target women on the BD or MDD spectrum. The purpose of this qualitative study was to learn more about the treatment needs and group experiences of women with problematic menopause symptoms and diagnosed on the BD and MDD spectrum, who participated in a CBGT intervention for menopausal symptoms.

***Methods:*** Narrative data recorded by clinicians (Interventionists' notes) and participants (Evaluation of Groups Survey) were analyzed using content analyses.

***Results:*** Several themes emerged from (*n* = 11 BD; *n* = 48 MDD) what women wanted help with (specific symptoms and general aspects of menopause), what women liked about CBGT (specific and general aspects of the program), and changes needed in the CBGT intervention (things wished for and barriers that interfered with the program). The two diagnostic groups differed in their responses, although both groups identified content and delivery gaps they wished would be addressed. Specifically related to their diagnosis, women most commonly talked about problems with worsening mood and mood instability and multiple stressors interfering with their ability to follow through with the intervention.

***Conclusions:*** These findings can help refine existing CBGT protocols for women diagnosed on the BD and MDD spectrum seeking help for menopause symptoms.

***Trial Registry:*** Parent study ClinicalTrials.gov [identifier: NCT02860910].

## Introduction

Up to 15% of more than 20 million women experiencing menopause each year in the United States are also diagnosed with a mood disorder, including bipolar disorder (BD) and major depressive disorder (MDD).^1^ Quantitative^2,3^ and limited qualitative research^4,5^ indicate women diagnosed with a mood disorder might experience an increase in depressive, hypomanic, or manic symptoms during perimenopause and postmenopause.

Cognitive behavior therapy (CBT) is a well-established, evidence-based treatment for menopausal symptoms,^6,7^ BD, and MDD, individually and in a group setting.^8^ CBT is a form of talk therapy that uses behavioral and cognitive interventions to help patients develop ways to cope.^9^ Early data show cognitive behavioral group treatment (CBGT) is effective in women with a combination of menopause symptoms and MDD, but literature is limited.^10^

To address this gap, our team conducted a single group trial of CBGT for women with menopausal symptoms and BD or MDD.^11^ Of 59 participants, 18.6% (*n* = 11) had BD and 64.4% (*n* = 48) had MDD. This study used an established CBGT protocol developed by Hunter and Smith^12^ to treat breast cancer survivors with problematic menopause symptoms.

In the trial, we also collected information about women's treatment needs and group experiences. The purpose of this qualitative study was to learn more about the treatment needs and group experiences of women with BD and MDD to better understand if existing protocols were sufficiently robust and comprehensive in addressing their needs. We performed a content analysis using qualitative data from the parent study noted above.^11^ Content analysis is a qualitative data analysis method that organizes words and phrases into groups and categories.^13^ We expected our findings would help determine what intervention content should be revised and/or refined to meet the treatment needs for women diagnosed with BD or MDD and problematic menopause symptoms. Shedding light onto these processes could potentially reveal refinements to enhance CBGT's reach and efficacy for these populations.

## Methods

All women (*n* = 11 BD; *n* = 48 MDD) provided informed consent.^11^ Ethical approval was obtained from University Hospitals Cleveland Medical Center's Institutional Review Board (IRB number: 20200825). Mood disorders were verified using the Diagnostic and Statistical Manual of Mental Disorders: DSM-5TM, 5th ed.^14^

Narrative data came from two sources within the parent study. First, we included intake evaluation and session notes, referred to as Interventionist's notes (*n* = 11 BD and *n* = 48 MDD) written by a trained psychologist or fourth-year psychiatry resident. Before the screening visit, during the intake evaluation, these clinicians asked women standard questions about menopause-related symptoms and questions about reasons for wanting to participate in the parent study. During the CBGT sessions, the same clinicians also recorded participants' verbatim comments about the CBGT intervention, psychosocial stressors, and chief complaints of symptoms on a flip chart that was visible to the entire group. Participants could see what was recorded and provide corrections/clarification, if needed. These intake evaluation and session notes were similar because they were recorded by clinicians, reflected women's verbalizations, and were transcribed verbatim for analyses.

Second, study participants (*n* = 8 BD; *n* = 41 MDD) provided written responses on an Evaluation of Groups Survey (EOG).^6^ This survey is described in more detail in the parent study.^11^ Of relevance here, is that at the end of the study, and as part of the EOG survey, women wrote in answers regarding the perceived usefulness of the group sessions, what they found less useful, their main concerns, and suggestions for session improvements. We used these verbatim written comments in the analyses.

Two team members analyzed the narrative data (DYC, JSC) using standard content analytic procedures as described by Miles, Huberman, and Saldaña.^13^ Both authors read all available text units. One author then coded each relevant text unit with a label to capture its essential meaning, grouped together similar codes, and organized them into categories. Another author reviewed the coding and categories against the original text units. Both team members reached consensus on the final categories through discussion.

## Results

Characteristics of the BD and MDD group are shown in Table 1. Categories for the Intake/Interventionist's notes are discussed for each group followed by categories from the EOG (Table 2). Table 3 shows examples of text units by category, source, and diagnostic group for each research question. Only verbatim comments were put in quotes.

**Table 1.** Demographics

Variable	BD (*n* = 11)	MDD (*n* = 48)
Age [M ± SD (range)]	50.9 ± 4.7 (44.3–60.1)	54.6 ± 5.7 (43.9–65.6)
Years of education [M ± SD (range)]	15.0 ± 1.9 (12–18)	15.1 ± 2.3 (11–20)
Number of jobs held in the past 5 years [M ± SD (range)]	1.2 ± 1.2 (0–3)	1.3 ± 1.0 (0–4)
Race [*n* (%)]		
Black or African American	7 (64)	27 (56)
White or Caucasian	4 (36)	21 (44)
Marital status [*n* (%)]		
Single, never married	3 (27)	10 (21)
Married	3 (27)	21 (44)
Separated	0 (0)	0 (0)
Divorced	5 (45)	16 (33)
Widowed	0 (0)	1 (2)
Highest level of education [*n* (%)]		
Junior high school	0 (0)	0 (0)
Some high school	0 (0)	2 (4)
Completed high school/G.E.D.	2 (18)	7 (15)
Some college, technical, or business school	4 (36)	18 (38)
Completed college, technical, or business school	4 (36)	10 (21)
Some graduate school	0 (0)	3 (6)
Completed graduate school	1 (9)	8 (17)
Employment status [*n* (%)]		
Unemployed	0 (0)	1 (2)
Homemaker	0 (0)	7 (15)
Employed part time	4 (36)	6 (13)
Employed full time	3 (27)	24 (50)
Retired	0 (0)	6 (13)
Disabled	4 (36)	4 (8)
Menopause status [*n* (%)]		
Postmenopause	9 (82)	34 (71)
Perimenopause	2 (12)	14 (29)
Generalized anxiety disorder [% yes]	7 (64)	31 (65)
SIGHA score [M ± SD (range)]		
Baseline	16.4 ± 7.9 (7–32)	15.5 ± 6.0 (5–32)
End of study	13.0 ± 10.2 (2–32)	12.1 ± 7.2 (1–26)

BD, bipolar disorder; M, mean; MDD, major depressive disorder; SD, standard deviation.

**Table 2.** Narrative Differences by Source and Group for Each Research Question Results Category

		Source	
Category	Subcategory	Interventionists’ notes (BD only, MDD only, **Both**)	EOG survey (BD only, MDD only, **Both**)
**RQ1: What are the treatment needs?**			
Managing specific symptoms	Mood	**Mood** **Mood instability** **Mood worsening** **Mood effects (on family or isolation)** *Mood improvement* Depressive symptoms Coping with mood (depression, mood instability) Managing frustration Managing guilt	**Mood** Mood worsening
	Anxiety	**Anxiety** Anxiety worsening Anxiety affecting mood, menopause Family members health affecting anxiety	
	VMS	**VMS** VMS worsening VMS worsening around menstrual cycle VMS triggers **VMS management**	VMS VMS management
	Sleep	**Sleep disturbance/SD—general** SD due to cognition (mind racing, stress, anxiety, rumination) SD due to physical sx (**VMS**, pain) SD due to both cognitive and physical causes	**Sleep disturbance** Sleep maintenance
	Cognitive	**Cognitive changes**	**Cognitive changes** Cognitive problems
	Physical	**Physical changes** Physical symptoms **Physical changes—aging** Learn about physical changes Reproductive aging	Physical changes—bodyweight Role changes—aging Physical changes—sexual behavior Physical changes—dry eyes
Learning about menopause and menopause management		**Menopause education** Menopause management Menopause and mood management Menopause and medical symptom management	
**RQ2: What are the CBGT experiences?** Benefits:			
General aspects		Reconnecting with self General learning	
Specific content		Education about symptoms—VMS, mood, sleep, physical symptoms **Education about VMS management** Education about coping Education on self-care Self-care	Normalizing/reassuring information Education/enlightenment Coping Self-care Education about managing symptoms (VMS, sleep/sleep problems, anxiety) **Identifying triggers** **Facts and myths**
Specific tools		Sleep diary CD Meditation Paced breathing/breathing Relaxation Managing negative thoughts Learning to pace self	**CD** Meditation **Paced breathing/breathing** Relaxation Sleep education CBT Learning to pace self
Feeling not alone		Not alone	Not alone **Shared experiences** Helpful validation Belongingness Connection Emotional support
Group support			Discussions Genuine conversations Emotional support *Other women encouraging other women* *“other women's opinions and experiences”* Sharing strategies Symptom management
Milieu			Confidential space Genuineness Nonjudgmental environment Felt valued *Different attitude*
**RQ3: What intervention content should be revised/refined?** Barriers:			
Stress		**General stress** Stress and loss/bereavement **Caregiver/relationship stress** Home-related stress **Financial stress** Education-related stress **Health-related stress** **Work-related stress**	General stress Financial stress Caregiver/relationship stress Health-related stress
To using CD			Time management Discipline Organization CD voice irritating *Home environment and other environmental factors* *Forgetfulness/distractions* *Fatigue*
To self-care		*Health-related problems* Caretaking	Time management
To using relaxation techniques		Home environment	Discipline
To using paced breathing			Time management Forgetfulness
Lack of program flexibility			The strict protocol Few choices of day of the week and time
Engagement			Repetitiveness of information
Group management/milieu			Timeliness of group sessions
**RQ4: What did women need, but did not receive?** Things wished for:			
CBGT maintenance			Ongoing menopause support and education
CBGT session content			Use of CD during every session Additional menopause education Practicing paced breathing together during every group More focus on mood disorders and the effect on menopause symptoms
CBGT milieu			More frequent group sessions More than 4 participants in a group Coping with uncomfortable information Have other women in the same stage of menopause in the same group
CBGT tools			Optional relaxation techniques **Digital version of the CD**
Other			Continuing menopause research Menopause education for providers

CD, Compact Disc; CBGT, cognitive behavioral group therapy; CBT, cognitive behavior therapy; EOG, Evaluation of Groups Survey; VMS, vasomotor symptoms.

**Table 3.** Example Text Units by Category, Source, and Group for Each Research Question

		Source	
Category	Subcategory	Interventionists' notes (BD only, MDD only, **Both**)	EOG survey (BD only, MDD only, **Both**)
**RQ1: What are the treatment needs?**			
Managing specific symptoms	Mood	Would like help with menopause and the depression… “I don't know what to do or how to handle it”	*“My irritability”* is a main issue or concern.
	Anxiety	“I was going through the menopause transition and noticed changes of increased anxiety.”	
	VMS	*Would like help with VMS management… She needs to take her clothes off at night because of feeling hot.*	“Hoping I don't panic if the hot flush is too bad” is a main issue or concern.
	Sleep	*She reported she can't sleep without a fan. She needs to readjust the fan because she is very hot.*	*“My sleep being very inconsistent in terms of very different wake and bed-times” is a main issue or concern.*
	Cognitive	Would like help with… “forgetfulness and difficulty with word finding. Trouble remembering people's name haven't seen in a while. Words and phrases are jumbled.”	“Forgetting names, dates, aspects of conversations, and having to search a long time to find objects such as cell phone, books, car keys, or any small thing that I lay down” are main issues or concerns.
	Physical	She reported vaginal dryness since menopause.	“Weight gain” is a main issue or concern.
Learning about menopause and menopause management		*She would like help with learning about the changes that her body is going to go through as she gets older.*	
**RQ2: What are the CBGT experiences?** Benefits:			
General aspects		“Just being here each week figuring things out and tweaking things” was helpful.	
Specific content		What she found helpful was knowing about other things that are a part of menopause: the depression and mood swings.	“It is not the end of the world. The symptoms can be managed.”
Specific tools		Her “well-being goals and the meditation have been particularly helpful in managing [her] anxiety and menopause symptoms.”	*Paced breathing “helped me to calm down and to focus and remain in the moment.”*
Feeling not alone		What she found helpful was: “Knowing it's happening to everyone. I felt like I was being attacked by hot flashes. It was tormenting me. It's natural, not something I did or didn't do.”	*“It [the group sessions] was very helpful because I got to see other people were going through the same thing that I was going through.”*
Group support			“The group openly discussed very private thoughts which helped me to understand my own insecurities with menopause.”
Milieu			It was “rewarding to talk things out with other people in a non-judgmental environment.”
**RQ3: What intervention content should be revised/refined?** Barriers:			
Stress		*She reported being stressed due to a car accident her husband got into and stress from being overwhelmed at work for the past 2 weeks.*	“Increased stress due to life situations, always fatigued and concentration issues, lack of feeling pleasure” are main issues or concerns.
To using CD			*The CD “would have been more useful if I could remember to listen to it.”*
To self-care		*She reported chronic stress and medical problems make it difficult to take time to care for herself.*	“It sounds a bit ridiculous, but sometimes it's hard to find even a few minutes for myself.”
To using relaxation techniques		*She mentioned that the main barriers to doing the daily relaxation were distractions in the home that don't allow for a quiet place to relax.*	“Maintaining the discipline to continue the relaxation techniques, meditating, awareness of thoughts, and diet” are concerns.
To using paced breathing			“It is always difficult to find the time” for paced breathing.
Lack of program flexibility			The least helpful aspect of the program was “the strict protocol of 6 weekly sessions. No ability to have flexibility.”
Engagement			The least helpful aspect was “some of the repetition of information.”
Group management/milieu			“Group started late and ran late. This was stressful for me after working all day.”
**RQ4: What did women need, but did not receive?** Things wished for:			
CBGT maintenance			“Are there any online forums/groups where women support each other in making self-care a priority?”
CBGT session content			*“I wish there was more focus on my mood disorder and its effect on my menopause symptoms.”*
CBGT milieu			“I wish we could have met twice per week but that probably isn't feasible.”
CBGT tools			*The relaxation CD “was more effective when I could use it on my phone as an mp3 file.”*
Other			“Continuity and advancement of this work is imperative to women and girl's health and well-being. It has been true for myself and I want the gift of finding out about it and participating historically in this new millennium.”

Only verbatim comments were put in quotes.

### Categories from interventionists' notes

#### Research question 1: What are the treatment needs for women with problematic menopause symptoms and BD or MDD?

Two categories of women's treatment needs emerged (managing specific symptoms and learning about menopause and menopause management).

##### Managing specific symptoms

At intake and during group sessions, women reported wanting help with managing specific symptoms that fell into six categories: mood, vasomotor symptoms (VMS), sleep disturbance, physical symptoms, cognitive problems, and anxiety.

###### Mood

For mood, women in both groups reported wanting help with managing mood, mood instability, mood worsening, and mood effects on family. Women talked about “mood,” “depression,” “feeling blah,” “feeling sad,” or “irritability.” Mood worsening included increased depression or more difficult depressive episodes. Mood instability included increased mood swings, mood swings due to hormone fluctuations, and “feeling like I'm going crazy.” Some women were concerned about how worsening moods affected their families and led to feelings of guilt or interpersonal conflicts, such as becoming more argumentative or short with others.

There were some differences in how women talked about mood. Women in the BD group talked about racing thoughts and flight of ideas improving since the onset of menopause. Women in the MD group reported wanting help coping with depressive symptoms and coping with mood, such as learning how to de-escalate when angry. Women with MDD expressed frustration with their change in memory, as well as guilt about not being able to help family members.

Women in both groups wanted help with managing anxiety. Women in the MDD group reported noticing worsening anxiety since perimenopause, after a hysterectomy, and/or during the menopause transition. They reported that moods and menopause symptoms were affected by their anxieties. In addition, women in the MDD group reported feeling worried about the health problems of family members and about being the caretaker.

###### Vasomotor symptoms

Several women in both groups wanted help managing VMS, such as intense or excessive sweating. For example, one woman reported, “feeling ugly or gross” due to sweating. Some women wanted to know how to better cope with or how to decrease the intensity and frequency of hot flashes and night sweats. Some women wanted to learn about nonpharmacological treatment options. Women in the MDD group wanted help with managing worsening VMS, especially a week before beginning menses and wanted to learn to manage hot flash triggers, such as VMS worsening during the summer months or due to stress.

###### Sleep

Women in both groups wanted help with managing sleep in general or specific aspects of sleep, such as help with managing sleep disturbances due to anxiety, or due to VMS. Some women in the MDD group wanted help with managing sleep disturbance due to stress, rumination, or racing thoughts and others due to pain. Some women in the MDD group wanted help managing sleep disturbances related to rumination along with chronic pain.

###### Cognition

Both groups talked about cognitive changes, including more forgetfulness, the need to look for words, and to rely on more lists. One woman in the BD group reported growing frustration with “words on the tip of her tongue.” Some women in the MDD group also reported difficulty remembering appointments, forgetting names of people, and feeling like their brains were in a fog.

###### Physical changes

Women in both groups wanted help with managing physical changes, such as weight gain and weight distribution in the mid-section, vaginal dryness, painful sex, and thinning hair. Women in the BD group reported having difficulty with the topic of aging coming up in social settings. Women in the MDD group reported difficulty embracing the aging process. Some women in the MDD group talked about the stress of not being able to have children and needing to accept this fact.

##### Learning about menopause and menopause management

Both groups wanted general education about how to manage menopause. Women in the BD group wanted to learn how to manage menopause and medical issues. Women in the MDD group wanted to learn about menopause management and education to understand what is and is not normal about menopause. They also wanted to know how to manage mood and menopause symptoms. For instance, one woman said she thought menopause was associated with “mature raging maniacs.”

#### Research question 2: What are the CBGT experiences for women with problematic menopause symptoms and BD versus MDD?

Four categories emerged about CBGT experiences that were beneficial (general aspects, specific content, specific tools, and not feeling alone).

##### General aspects

Women in the MDD group found general aspects of the CBGT beneficial. Few women were able to reconnect with themselves, referred to as getting their old selves back. For women with MDD, learning general aspects about menopause helped them figure things out and solve problems.

##### Specific content

Learning how to manage VMS was beneficial for women in both groups. Only women in the MDD group said that information they gained through education about VMS, mood, sleep, and physical symptoms was beneficial. Education also helped women cope with mood changes accompanying the menopausal transition, realizing that “I know I am not crazy.” In addition, education about the importance of self-care and implementing self-care goals was also useful.

##### Specific tools

Women in the BD group did not describe any benefit related to the tools at intake or during group sessions. Women in the MDD group described benefits from the following: the sleep diary, the Compact Disc (CD), meditation skills, paced breathing and breathing exercises in general, relaxation, learning to pace themselves, and managing negative thoughts. For instance, one woman stated that following her goals, pacing herself, and the relaxation techniques had been helping her manage her stress and reduce the impact of bothersome hot flashes.

##### Feeling not alone

One woman in the MDD group reported that not “feeling alone” was beneficial. She felt better simply knowing that hot flashes were happening to everyone.

#### Research question 3: What intervention content should be revised and/or refined to meet the treatment needs for women with problematic menopause symptoms and BD or MDD?

Three categories emerged as barriers (stress, self-care, and using relaxation techniques).

##### Barriers—stress

Both groups reported instances where general, caregiver and relationship, financial, and health- and work-related stresses hindered their ability to participate in the CBGT intervention assignments and/or group sessions. General stress was described as having problems with stress or being overwhelmed. Caregiver and relationship stress resulted from caring for, or relationship problems with, family members. Women in both groups reported instances of stress related to employment and finances that led to interpersonal conflict. As barriers to the intervention, the MDD group cited stress from loss and bereavement, stress related to education/going back to school, and stressful home environments, such as adult children moving back home. Women in the MDD group mentioned stress associated with chronic pain, discomfort in their joints, fatigue, and fibroids on their bladder.

##### Barriers to self-care

Women in the BD group said health-related problems were barriers to self-care, and women in the MDD group reported that the need to take care of others posed barriers to self-care.

##### Barriers to using relaxation techniques

Women in the BD group reported their home environment did not provide a place to relax, which was a barrier to using the relaxation techniques adequately.

#### Research question 4: What did women need, but not receive in the CBGT intervention?

There were no data to report for this question from the Intake and/or group sessions.

### Categories from the EOG

#### Research question 1: What are the treatment needs for women with problematic menopause symptoms and BD versus MDD?

One category emerged for women's treatment needs (managing specific symptoms).

##### Managing specific symptoms

On the EOG survey, women in both groups wanted help with mood, sleep disturbance, and cognitive changes. In addition, women in the MDD group talked about wanting help with mood worsening, managing VMS, sleep maintenance, cognitive problems, and physical changes. Women in the MDD group talked about “forgetting names, having to search a long time to find objects” and “wanting to get solid sleep without sweats.” The MDD group also talked about wanting help for increased bodyweight, changes in sexual behavior, and “dry eyes.” One woman said she wanted help “coming to terms with my kids not needing as much anymore.”

#### Research question 2: What are the CBGT experiences for women with problematic menopause symptoms and BD versus MDD?

Five categories for CBGT experiences were beneficial (specific content, specific tools, not feeling alone, group support, and the group milieu).

##### Specific content

Both groups talked about identifying triggers “that can make hot flashes worse” and “finding out facts and myths of menopause” as helpful. Women in the MDD group wrote about finding the following content beneficial: normalizing and providing reassuring information, education that was enlightening, coping, self-care, and education about managing VMS, sleep, and anxiety symptoms. For example, women talked about “the information being reassuring,” and knowing “things happening to my body was normal” as beneficial.

##### Specific tools

Women in both groups talked about the CD and paced breathing and other breathing exercises being beneficial. For example, the paced breathing helped women “to calm down,” “alleviate frustration,” and “to focus on abdominal breathing.” Women in the MDD group talked about the meditation, relaxation techniques, sleep education, and CBT being helpful. Women said “guided meditation was calming,” and learning about “my sleep issues” was beneficial.

##### Feeling not alone

Women in both groups said they found it beneficial to know they were “not alone in my feelings about menopause” and had shared experiences with other women. Women in the MDD group noted that helpful validation, emotional support, belongingness, and connection were beneficial. For example, women “felt comraderies” liked “meeting new people,” and “felt heard and understood.”

##### Group support

About group support, women talked differently. BD participants mentioned benefits of “other women encouraging other women” and learning about “other women's opinions and experiences.” MDD women mentioned group support benefits in terms of discussions, genuine conversations, emotional support, and sharing strategies for managing “feeling helpless against the sweating,” “feeling more in control of my symptoms,” and being “able to pace myself better.”

##### Group milieu

Women in the BD group expressed that a benefit of the group milieu was having their attitudes regarding menopause and hot flashes shifted from “feeling shameful” to “seeming normal.” MDD women appreciated the confidential space, genuineness of group participants, the nonjudgmental environment, and feeling valued.

#### Research question 3: What intervention content should be revised and/or refined to meet the treatment needs for women with problematic menopause symptoms and BD or MDD?

Eight categories emerged as barriers to the intervention (stress, using the CD, self-care, relaxation techniques, paced breathing, lack of program flexibility, engagement, and the group milieu).

##### Barriers—stress

The MDD group said general, financial, caregiver/relationship, and health-related stress were barriers to the intervention, such as “general tiredness,” “always fatigued,” and “increased stress due to life situations.”

##### Barriers to using the CD

Both groups talked about barriers to using the CD. BD women included the home environment, such as listening to it “in bed and falling asleep within a few minutes” and other environmental factors, such as trying “to relax while driving” as barriers. Other barriers were forgetfulness, being easily distracted, and fatigue. “Finding it hard to schedule it in” and “fitting into my routine,” lack of discipline and organization, and finding the CD “voice irritating” and “somewhat intrusive” were barriers to using the CD for MDD group.

##### Barriers to self-care

The MDD group talked about time management being a barrier to self-care, such as “finding time to take care of myself.”

##### Barriers to relaxation techniques

The MDD group talked about discipline being barriers to using relaxation techniques, preferring “other relaxation techniques” and “maintaining discipline to continue” practicing relaxation techniques.

##### Barriers to using paced breathing

The MDD group talked about time management, forgetfulness, or forgetfulness due to seeking immediate relief as barriers to using paced breathing. One woman said she “would think of the fan for immediate relief.”

##### Lack of program flexibility

Women in the MDD group talked about the lack of flexibility, such as the strict schedule of six weekly consecutive group sessions made joining the sessions more stressful for some participants.

##### Barriers to engagement

The MDD group talked about how the “repetitiveness of the information” provided was a barrier to engagement, but since “it might help others; it was not really a problem.”

##### Problems managing the group/milieu

Some women in the MDD group were frustrated by the facilitator's inadequate management of the group and milieu. For example, it was frustrating when the facilitator would wait for participants who were running late from work to arrive and then allow group sessions to run past the scheduled time.

#### Research question 4: What did women need, but did not receive in the CBGT intervention?

One category emerged for what women wanted, but did not receive (things wished for).

##### Things wished for

Women in the BD group described things they wished for, such as to “practice paced breathing together in every group” and “more focus on mood disorders and their effect on menopause symptoms.” Women in the MDD group talked about wanting to use the CD during every session, wanting ongoing menopause support and education, and specific aspects of the milieu and tools they wanted changed. For example, some women wanted more frequent group sessions, some wanted more women in each group, and some wanted women in the same stage of menopause in each group. Other women in the MDD group wanted optional relaxation techniques. Women in both groups wanted a digital version of the CD.

## Discussion

We sought to uncover specific needs of women with BD or MDD participating in a CBGT intervention to manage problematic menopausal symptoms. The first major finding was the group difference in the amount of information women provided on the EOG survey. Women in the BD group offered fewer comments overall. It is not clear whether they wrote fewer comments because they were less engaged, perceived fewer benefits, or just had less to say about the CBGT intervention.

A second major finding was that CBGT intervention manuals may need to be revised to fulfill the needs of these populations. For example, some information is missing from Hunter and Smith's CBGT and Green's CBT-Meno manualized treatment protocols, both of which have been shown to reduce mood and menopausal symptoms.^12,15^

Our findings suggest adding content on worsening mood may be beneficial when CBGT is used to treat problematic menopause symptoms in women with BD and/or MDD. Although the CBT-Meno workbook seeks to provide several interventions to help with mood, anxiety, and stress, authors state that if readers are experiencing clinical depression or had a previous anxiety disorder, they should seek additional help.^15^ Worsening of mood, mood instability, more debilitating depression since menopause, and anxiety were noticed in both groups. These findings are similar to other reports.^16^ In Sajatovic et al., women with schizophrenia, BD, or MDD (*n* = 39) reported that menopause affected the emotional and mental well-being of themselves and their families.^17^ Similarly, in a qualitative study by Freeman et al., women reported an increase in irritability, depression, hypomanic symptoms, and mood cycling during menopause.^18^ Some symptoms participants experienced during menopause might be seen as precipitants to a mood disorder relapse, without considering hormonal fluctuations as a precipitating factor that could be treated with a menopause CBGT protocol as part of an integrated treatment.

Additional content on overcoming barriers to using skills learned may be beneficial for this population. Participants in both groups said multiple stressors interfered with following prescribed assignments from the CBGT intervention. Our findings are supported by Friedman et al., who found that women with BD, schizophrenia/schizoaffective disorder, and MDD (*n* = 91) had a relatively high vulnerability to psychosocial problems and menopause symptoms.^19^ The multiplicity of stressors for these populations will likely need to be addressed through extra time or an additional module within the CBGT intervention.

Although the parent study followed the planning, prioritizing, and problem-solving protocol by Hunter and Smith,^12^ our study population noted additional barriers to practicing assigned skills. The CBGT interventions from the parent study^11^ did not address additional barriers noted by participants, such as forgetfulness, becoming easily distracted by other activities, difficulty finding a quiet place at home, and lack of discipline. It might be beneficial to anticipate these additional barriers upfront to increase adherence to the CBGT intervention. Improving adherence could help participants benefit more fully from the intervention.

Recognizing that women with mood disorders have the burden of managing multiple areas of their lives (i.e., their mood disorder, menopause symptoms, and multiple stressors) and addressing this burden during group therapy might prove beneficial. Women experience multiple menopausal symptoms.^20,21^ In other populations experiencing multiple symptoms, such as cancer patients, symptoms can be synergistic or multiplicative and not just additive.^22,23^ For example, four symptoms are more than four times the burden of one symptom. The same may be true for women at menopause. For women with BD and MDD already experiencing mood symptoms, the additional changes from menopause (VMS, sleep, and cognitive) may serve as tipping points where symptoms exceed coping resources.

Learning that other women experienced similar menopause symptoms, mood symptoms, and physical and cognitive changes helped to normalize women's experiences, confirming previous studies.^6,24^ However, the request for ongoing support, more research studies, and more frequent CBGT intervention sessions was unique to the MDD group and practicing relaxation together was unique to the BD group. Both groups mentioned several benefits of being part of a supportive group, which suggests that delivering this treatment in other formats, such as a self-help workbook, may not be as beneficial.

A study limitation was the small sample size of eight EOG surveys for the BD group. Typically, sample sizes between 10 and 20 are used in qualitative research to achieve saturation. Another potential limitation stemmed from the Interventionists' notes. Intake and session notes may have been subjectively edited by the clinicians as they were being written from participants' verbatim descriptions and/or descriptions recorded on the flip charts. Despite these limitations, findings provide a preliminary understanding of what women liked about CBGT, what kind of help they wanted, and potential intervention changes needed for women diagnosed with problematic menopause symptoms and BD or MDD.

## Conclusions

Published, evidence-informed CBGT manuals to help women manage menopause symptoms^12,15^ and an internet-based CBT guide to help breast cancer survivors manage menopause symptoms induced by cancer treatments^25^ are not specific to women diagnosed with BD or MDD. In addition, women with BD may be excluded from trials.^6,7,10,12,15,24,25^ In the Meno-CBT trial,^10^ although 70% of intervention group participants (*n* = 26) were diagnosed with major depressive or persistent depressive disorder, no qualitative data from these women were presented. In addition, women with BD were excluded.

Existing CBT treatments^12,15,25^ have not addressed: education regarding how mood symptoms affect menopause for women diagnosed with BD, distinguishing menopause symptoms from bipolar symptoms, multiple barriers to engage in treatment, and coping with multiple stressors. In addition, although education was offered, there were no specific interventions targeted to address weight issues or problems with memory. Our findings indicate that these existing interventions need to be tailored for women diagnosed with BD and MDD to fully address their needs.

Based on our findings, we recommend two pathways forward for research. First, a descriptive survey could be done to gather more information about how these populations experience menopause and their perceived treatment needs. These survey results could provide further information about tailoring existing CBGT interventions for these populations. Second, a refined or tailored intervention protocol should be developed and tested in these populations. Advancing research in this area could help improve delivery of services for women with problematic menopause symptoms and BD or MDD.

## Abbreviations Used

BD: bipolar disorder
CBGT: cognitive behavioral group treatment
CBT: cognitive behavior therapy
CD: Compact Disc
EOG: Evaluation of Groups Survey
MDD: major depressive disorder
SD: standard deviation
VMS: vasomotor symptoms
